# Supplementary figures and images for: Genotype-phenotype characterization and functional reconstitution of pathogenic β-catenin variants from CTNNB1 syndrome patients
Source: PLoS Genet. 2025 Oct 13;21(10):e1011907. doi: 10.1371/journal.pgen.1011907 (PMC12543288; doi:10.1371/journal.pgen.1011907)

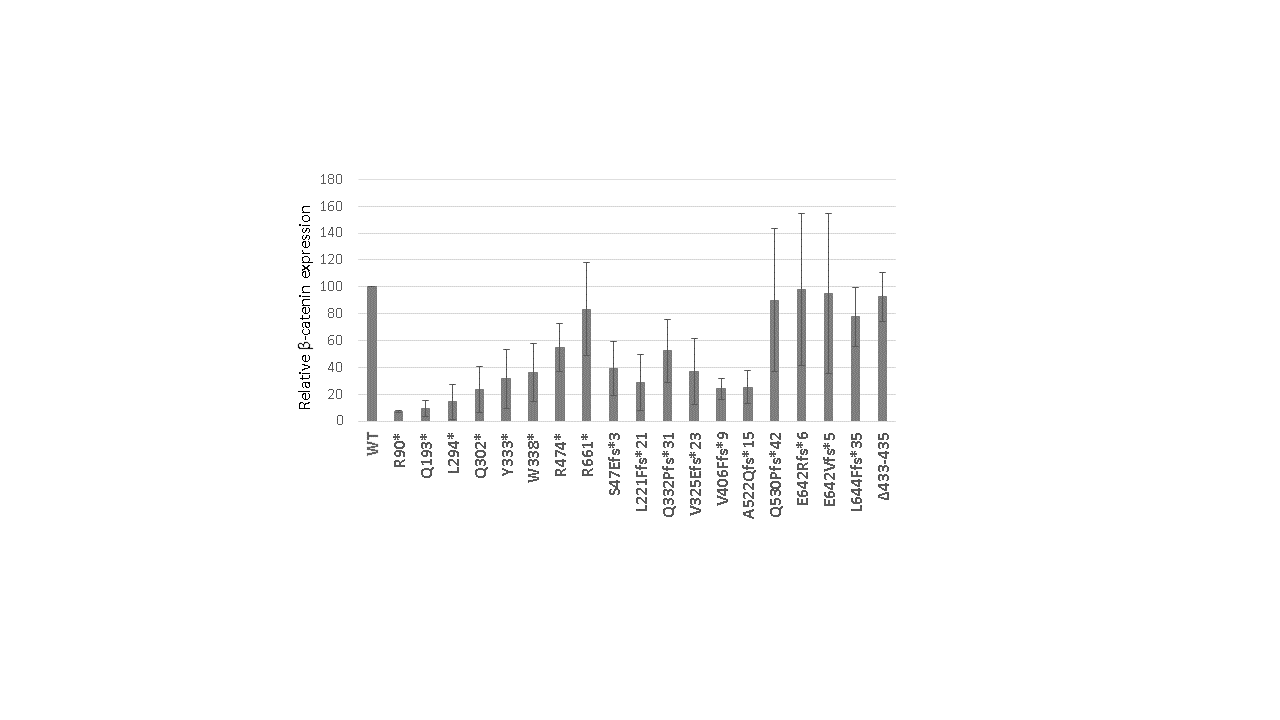

Supplement: S1 Fig — COS-7 cells were transfected with pRK5 mammalian expression plasmids encoding the indicated β-catenin variants and processed for immunoblot as in Fig 2, using anti-β-catenin N-terminal or C-terminal antibodies. Protein bands were quantified and represented with respect to β-catenin wild type. Data are shown as relative expression ± SD, from at least three independent experiments. (PNG) [file pgen.1011907.s001.png]
