# Supplementary material for: Genotype-phenotype characterization and functional reconstitution of pathogenic β-catenin variants from CTNNB1 syndrome patients
Source: PLoS Genet. 2025 Oct 13;21(10):e1011907. doi: 10.1371/journal.pgen.1011907 (PMC12543288; doi:10.1371/journal.pgen.1011907)
Supplement: S1 Table — (PDF) [file pgen.1011907.s002.pdf]

Table S1: Patient classification by genotype groups			
Patient number	High (H) or Low (L) protein stability	Residual transcriptional activity	Reinitiation of translation
23	H	+	+
3	H	-	+
6	L	-	-
25	L	-	-
11	L	-	-
19	L	-	-
7	L	+	-
1	H	-	-
14,21	H	+	+
20	L	-	-
12	L	-	-
8	L	-	-
4	L	-	-
10	H	+	-
9	H	+	-
5	H	-	-
22	H	-	-
15	H	-	-
18	H	+	-
17	L	-	-
24	H	-	-
16	H	-	-
13	L	-	-
2	L	-	-
