# Supplementary material for: Genotype-phenotype characterization and functional reconstitution of pathogenic β-catenin variants from CTNNB1 syndrome patients
Source: PLoS Genet. 2025 Oct 13;21(10):e1011907. doi: 10.1371/journal.pgen.1011907 (PMC12543288; doi:10.1371/journal.pgen.1011907)
Supplement: S2 Table — (PDF) [file pgen.1011907.s003.pdf]

Table S2: Clinical, psychological and cognitive domains differences between patient groups						
	Protein stability		Residual transcriptional activity		Reinitiation of translation	
	U	*p*	*U*	*p*	*U*	*p*
Clinical variables						
Sleep disturbance scale for children	88	0.611	65	0.929	56.5	0.295
First word	79	0.956	50	0.458	52	0.496
VSS	65.5	0.406	49.5	0.423	43	0.938
CFCS	83	0.810	56	0.701	29	0.369
EDACS	81.5	0.852	59.5	0.836	54	0.409
MACS	92	0.470	45	0.297	38.5	0.803
Autism						
SCQ	67	0.574	59	0.836	46.5	0.748
Social Relating (SCQ)	75	0.894	68.5	0.745	55	0.369
Communication (SCQ)	77	0.979	56.5	0.701	49	0.642
Range of interest (SCQ)	58	0.295	47	0.357	38	0.803
CARS	74	0.852	77.5	0.389	53	0.452
Behavioral problems						
Internalizing problems (Vineland-3)	63	0.437	68	0.790	48.5	0.642
Externalizing problems (Vineland-3)	72.5	0.764	89	0.125	46.5	0.748
Maladaptive behaviors (Vineland-3)	77.5	0.979	66.5	0.836	42	1
Motor functioning						
GMFM88	40	0.203			20	0.195
Lying & rolling (GMFM88)	47	0.418	43.5	0.353	26	0.434
Sitting (GMFM88)	46	0.381	50.5	0.891	22	0.262
Crawling & Kneeling (GMFM88)	35.5	0.107	50	0.891	23.5	0.300
Standing (GMFM88)	43.5	0.283	56	0.837	22	0.262
Walking, running & jumping (GMFM88)	40.5	0.203	45.5	0.630	30	0.652
Adaptive functioning						
Total Vineland-3	96	0.347	**96**	**0.047***	52.5	0.452
Receptive (Vineland-3)	51	0.152	60.5	0.883	35	0.642
Expressive (Vineland-3)	76.5	0.936	70.5	0.657	37	0.748
Written (Vineland-3)	**40**	**0.040***	63.5	1.0	28.5	0.331
Personal (Vineland-3)	47.50	0.098	62.5	0.976	28.5	0.331
Domestic (Vineland-3)	**39.5**	**0.035***	50.5	0.458	26	0.262
Community (Vineland-3)	**38**	**0.030***	65	0.929	22	0.154
Interpersonal relationship (Vineland-3)	63.5	0.437	67.5	0.790	29.5	0.369
Play and leisure (Vineland-3)	88	0.611	63.5	1.0	36.5	0.695
Coping skills (Vineland-3)	61	0.376	60	0.883	29.5	0.369
Cognitive functioning						
Comprehension of instructions (NEPSY-II)	46	0.150	69.5	0.524	32.5	0.575
Peabody	48.5	0.186	53	0.710	30	0.477
Matrices (WNV)	**27**	**0.009***	45.5	0.383	32	0.575
Object assembly (WNV)	**25.5**	**0.006***	50	0.576	42	0.911
Recognition (WNV)	47	0.167	45	0.383	24.5	0.241
Boston Naming Test	25.5	0.321	25.5	0.956	17.5	0.676

CARS: Childhood Autism Rating Scale; CFCS: Communications Function Classification System; EDACS: Eating and Drinking Ability Classification System; GMFM88: Gross Motor Functioning Measure 88; MACS; Manual ability Classification System; SCQ: Social Communication Questionnaire; VSS: Viking Speech Scale; WNV: Wechsler Non Verbal.
