# Supplementary material for: Genotype-phenotype characterization and functional reconstitution of pathogenic β-catenin variants from CTNNB1 syndrome patients
Source: PLoS Genet. 2025 Oct 13;21(10):e1011907. doi: 10.1371/journal.pgen.1011907 (PMC12543288; doi:10.1371/journal.pgen.1011907)
Supplement: Attachment — Submitted filename: Response to Reviewers.23jul25.pdf [file pgen.1011907.s006.pdf]

Caroline E. Nunes-Xavier, Rafael Pulido
Immuno-Oncology Group, Biomarkers in Cancer Group
Biobizkaia Health Research Institute
Barakaldo, Spain

PLOS Genetics Editors 23 July, 2025

Dear PLOS Genetics Editors,

Thank you very much for your letter from 15-May-2025 regarding our Manuscript PGENETICS-D-25-00398, and for your invitation to submit a revised version of the manuscript addressing the points raised during the review process. We have carefully revised the manuscript following the editor's and reviewer's comments and recommendations, and below are our responses to their specific points. Please note that we have added two supplementary tables (Table S1, Table S2) and some new items to former Figures 3 and 4 (new Figure 3C-D and Figure 4D), and we have re-organized and modify the figures according to the suggestions from the reviewers (former Figures 2 and 3 have been merged in a new Figure 2). In addition, since new information has been included in the manuscript, some new references have been added. Finally, due to the new set of results incorporated into the manuscript, we have added Ana Rodríguez-Ramos as a new co-author of it, and the appropriate form has been submitted together with the revised manuscript.

Please find below our answers, point by point, to the reviewers comments:

Reviewer #1:

We thank the reviewer for her/his time and supporting and inspiring comments. Please find below our point to point answers to the specific comments.

- Comment: *The authors report significant differences in only two items (out of six) of the Wechsler Nonverbal Scale of Ability between two groups of patients clustered according to protein stability data. It is unclear whether the authors investigated differences between these two groups in other adaptive and neuropsychological assessments, and if so, whether any significant findings survived correction for multiple comparisons. The phenotypic protocol is only referenced via previous publications from the same group. Additionally, the lack of significant differences in other clinical variables assessed—such as expressive and receptive language, executive functions, adaptive functioning, and clinical manifestations—is only vaguely mentioned with reference to other "under review" papers. For the sake of scientific accuracy, the phenotypic features analyzed and compared between groups should be explicitly listed, and the statistical methods used should be reported, Furthermore, there appears to be no clear correlation between protein stability and residual transcriptional activity, with many variants classified as "highly stable" exhibiting apparently poor transcriptional activity. Why did the authors not explore whether residual transcriptional activity correlates with phenotype? It is unclear why the authors grouped patients solely based on protein stability, given that their own data (e.g., Figure 7) suggest that the reduced degradation of transcriptionally inactive proteoforms does not enhance their transcriptional function.*

In summary, to address these concerns, the authors should:
• Provide a detailed phenotypic classification of the cohort across all domains (motor, cognitive, adaptive);

- Our answer: **We thank the reviewer for these appropriate comments. We have investigated differences between the two stability groups in all the adaptive and neuropsychological assessments than were made in our two previous reports (Pallarés-Sastre et al. 2025a, PMID 40240530; Pallarés Sastre et al. 2025b, PMID 40145647; ref 32 and ref 33 in this manuscript), and relevant statistically significant differences were only found in the Matrices and Object assembly items. In addition, we have also searched for differences in groups according to other experimental criteria, including residual transcriptional activity and reinitiation of translation, and no other statistical differences that are not biased by the age of the patients were found (see also our next answer below). To make this clearer, and following the reviewer suggestion, we have added two supplementary tables: Table S1, that lists the distinct groups of patients according to the different experimental criteria; and Table S2, that summarizes all the clinical variables analysed, the name of the standardized tests, and the *U* and *p* statistical values for the comparisons between groups. The complete phenotypic protocols with a detailed explanation of each test used are available on the above indicated reports, which are now published and open-access available (ref 32 and 33). Finally, a novel Figure 3C-D has been added to illustrate that the residual transcriptional activity of some β-catenin C-terminal truncated variants is a bona-fide β-catenin transcriptional activity.**

- Comment: *• Justify their rationale for clustering variants based on protein stability (or consider re-clustering based on residual transcriptional activity, which may offer better external validity)*

- Our answer: **As mentioned in our previous answer, and following the reviewer consideration, we have re-analyzed our genotype-phenotype analysis based not only on protein stability, but also on residual transcriptional activity and on reinitiation of translation, and the results have been incorporated in the new supplementary Table S1 and supplementary Table S2. As shown, no relevant statistically significant differences were found between residual transcriptional activity groups or between reinitiation of translation groups. Total Vineland-3 score (ABC) was significantly different when comparing residual transcriptional activity groups, but this result may be biased by the difference in sample size (n= 7 vs n= 18) and patient age. This has been explained and justified in the manuscript.**

- Comment: *• Clearly report the statistical methods used in the genotype-phenotype analysis.*

- Our answer: **A more complete and detailed information has been added at the "Phenotypic and statistical analysis" subsection from "Materials and methods".**

- Comment: *FIGURE 3: A and B should be added to the figure.*

- Our answer: **Letters have been added to the former Figure 3. Please note that to facilitate the understanding of Figure 2 and Figure 3, these figures have been merged in a <u>new Figure 2</u> containing three panels: A, B. and C.**

Reviewer #2:

We thank the reviewer for her/his dedicated time and interesting and helpful comments. Please find below our point to point answers to the specific comments.

- Comment 1: *Throughout this paper there is minimal consideration given to the effects of non-sense mediated decay (NMD) elicited at the transcript level in the context of patient cell lines. If the message is degraded by NMD the relative stability of the predicted protein as determined by ectopic expression from a cDNA sequence is inconsequential.*
-Do the authors have access to patient cell lines or RNA extracted from blood where measurements of NMD could be made?
-The aminoglycoside (or alternative PTC read through approach) and protein stabilisation experiments would have much greater impact if performed on patient derived cell lines.

Our answer: **We agree with the reviewer on the importance of the potential effects of NMD on the stability of the CTNNB1 transcript variants from the reported patients, since most of them carry a premature termination codon. This issue was addressed in the Discussion of the former version of the manuscript, and we have now made more emphasis on it by including specific comments both in the Introduction and in the Discussion sections. In addition, we have added a column to <u>Table 1</u> indicating the distance of the PTC to the downstream exon-exon junction, which is indicative of the potential NMD degradation, and we have made mention to it in the Table 1 legend and in the text.**

We also agree with the reviewer on the convenience of performing RNA analysis from patient samples. Currently, we have no access to these samples, although the patients are in the process of being enrolled to donate biological samples, which will constitute the basis of our upcoming work on this area.

- Comment 2: *The N-terminally truncated forms of the protein that are formed from re-initiation of translation do not have the b-TrCP binding site. Even though the efficiency of translation re-initiation is low the overall stability of these proteins should no longer be dictated by the destruction complex.*
-The results of the cycloheximide block are at odds with this where there should be an expectation that CTNNB1 full-length protein should degrade rapidly in the absence of translation? It would be better to see these N-terminal truncations assessed by CHX chase as is shown in Fig 5B for p.Arg474Ter and p.Arg661Ter and / or inclusion of a p.Ser33Ala full length CTNNB1 which should remain stable for comparison to show that the destruction complex is active in the culture system in use.

BIOBIZKAIA
EUSKAL OSASUN IKERKUNTZA
INVESTIGACIÓN VASCA EN SALUD
BASQUE HEALTH RESEARCH

Our answer: **Folowing the reviewer suggestion, we have performed CHX chase experiments using the reinitiation of translation variants R90* and S47Efs*3, as well as a S33C full-length variant (<u>new Figure 4D</u>). The results show similar degradation rates for these variants, when compared to β-catenin wild-type.**

-Are there phenotypic differences specific to these patients compared to others with PTC variants that do not re-initiate?

Our answer: **We have also analysed phenotypic differences between the patients carrying reinitiation variants and the rest of patients, and found no statistical differences between these two groups. These results have been incorporated in the new <u>supplementary Table S2</u>.**

-Are these transcripts subject to NMD in patient cells or if unavailable when these variants are knocked into the CTNNB1 gene in a cell line?

Our answer: **As we mention above, the patient cells are not available at this moment. We present now in the manuscript (<u>Table 1</u>) a prediction of NMD sensitivity based on the relative location of the premature stop codon with regard to the downstream exon-exon junction, although we agree with the reviewer that this needs experimental verification. These considerations have been incorporated in the Discussion section of the manuscript.**

Comment 3*: Genotype to phenotype correlations made between high vs. low stability patients are very subtle and there are also outliers in the MDS plot.*

Our answer: **We have re-examined and repeated experiments to have a more robust classification of high vs. low stability patients, and the results are shown in the new <u>Figure 5</u>. As shown, a very good statistical significance is obtained in the two cognitive tests. There are still three outlier patients, and we believe that this could be due to the high patient clinical heterogeneity, which is a common feature of CTNNB1 syndrome and other neurodevelopmental syndromes. This is now mentioned in the revised manuscript.**

-What other covariates were considered especially e.g. biological sex, socio-economic status, underlying polygenic risk for low educational attainment?

Our answer: **More information related to the characteristics of the sample have been added in Table 1. However, socio-economic status and other variables were not asked to the sample and were not taken into consideration when carrying out the analyses. We prioritized the description and analyses of the phenotype including all the patients available, taking into consideration CTNNB1 syndrome is an ultra-rare disease, rather than analysing how other covariates could interfere results.**

-What is the significance for the patient for performing better or worse in these specific domains given many other clinical outcomes are equivalent?

Our answer: **Given the sample size and the fact that the aim of the study is not to determine the clinical significance of the CTNNB1 syndrome phenotype, the results suggest that performance on visuospatial tasks could be a distinguishing feature depending on variant protein stability. However, this is a preliminary finding based on a very small sample size and needs additional confirmation. This has been addressed in the Discussion section.**

Comment 5: *As the authors note aminoglycosides are not a feasible treatment however other compounds available such as PTC124 (Ataluren) could be used and is easily obtainable and is more effective. Why was this approach excluded?*

Our answer: **In our hands (as well as in other laboratories), the effectiveness of PTC124 as a readthrough inducer is very low. We ignore the reasons of these inter-laboratory discrepancies regarding PTC124, and we are actively searching for other non-toxic, more reliable, readthrough inducers.**

Comment 6: *In the discussion the authors discuss the efficacy of restoration of CTNNB1 by GSK3B. In the KO model, the mutant allele is completely non-functional and does not express Ctnnb1 so GSK3B inhibition is only protecting the wild type allele from degradation by the destruction complex not restoring the mutant allele. Looking only at transcriptional activity also disregards the role of CTNNB1 in cell-cell adhesion where C-terminal truncations may not form strong interactions with cadherins . There are some missense variants implicated in CTNNB1 syndrome that have no effect on the topflash assay compared to wild type CTNNB1, e.g. the recurrent p.Gly557Arg and also the Bfc mutation in mice is slightly gain of function in this assay. The risks of expressing truncated proteins that the NMD and unfolded protein responses have evolved to prevent should be discussed.*

Our answer: **We agree with the reviewer on the potential pathogenic importance of cell-cell adhesion interactions mediated by β-catenin variants, which is reinforced by the observation mentioned by the reviewer that some CTNNB1 missense mutations found in patients do not seem to affect β-catenin transcriptional activity. The study we present in our manuscript is focused on our cohort of Spanish CTNNB1 syndrome patients, where there are not missense CTNNB1 variants. Interestingly, we have found that some truncated variants from our cohort display a low but consistent transcriptional activity (Figure 2). We present now additional experiments using compound mutations with L338P, a patient-derived missense loss-of-transcriptional activity variant. The combination of the two mutations causes a stronger decrease in β-catenin transcriptional activity, suggesting that the residual activity of the truncated variants is bona-fide β-catenin transcriptional activity. This has been incorporated in the manuscript as a new Figure 3C-D, and described accordingly in Result and Discussion sections. The functional analysis of additional missense CTNNB1 variants will be addressed in our future studies widening the analysis of pathogenic CTNNB1 variants;**

nevertheless, since we find the observation regarding these missense variants of special interest, we have included some indications to it in the Discussion section.

Comment 7: *Throughout: Gene symbols for all species i.e. CTNNB1/Ctnnb1 should be in italics.*

Our answer: **Gene symbols have been written in italics.**

Comment 8: *Line 64-65: Check grammar: "The syndrome is characterized by a generalized delay in children development and shows a great phenotypic variety."*

Our answer: **The indicated sentence has been modified.**

Comment 9: *The use of X to denote a termination codon is incorrect. In the IUPAC code X means a non-specific amino acid. The correct single character code for a termination codon is * see https://hgvs-nomenclature.org/stable/recommendations/protein/substitution/ and https://iupac.qmul.ac.uk/AminoAcid/AA1n2.html*

Our answer: **Nomenclature of termination codon has been corrected following HGVS recommendations**

Comment 10: *Terminology switches between mutation and variant. Suggest to use the term variant for this work because it is more appropriate for human related literature.*

Our answer: **The term “mutation” has been changed by “variant” in all cases.**

Comment 11: The pRK5 plasmid is referred as described in ref 55 but that reference directs to an earlier one with the quote "Mammalian expression plasmid, pRK5 β-catenin, has been previously reported" Fuchs M, Müller T, Lerch MM, Ullrich A. J Biol Chem. 1996 Jul 12;271(28):16712-9.

Our answer: **The indicated reference (Fuchs et al., 1996) has been added to the manuscript.**

Reviewer #3:

We thank the reviewer for her/his dedicated time and supporting and helpful comments. Please find below our point to point answers to the specific comments.

Comment 1: *The analyses presented in Figures 5B, 7A, and 8B may not be optimal. Given the multiple conditions and variables tested, a two-way ANOVA would be more appropriate to evaluate potential interaction effects.*

Our answer: **As suggested, a two-way ANOVA has been made for former Figures 7A and 8B (current Figures 6A and 7B), and appropriate indications have been incorporated to the figures. Former Figure 5B (current Figure 4B) lacks variables to perform ANOVA test.**

Comment 2: *Statistical significance should be explicitly indicated within the figures, rather than described solely in the legends, as seen in Figure 6A.*

Our answer: **As suggested, statistical significance is now explicitly indicated within the figures.**

Comment 3: *The panels in Figure 3 should be clearly labeled as A and B to enhance interpretability.*

Our answer: **Panels have been labelled (see also next comment and answer).**

Comment 4: *Figures 2 and 3 appear to be conceptually linked, as Figure 3A summarizes data from Figure 2. Merging these into a single figure could improve logical flow and reduce redundancy.*

Our answer: **Many thanks for this suggestion. Figures 2 and 3 have been merged: Former Figure 2 is now Figure 2A, and former Figure 3 is now Figure 2B and 2C**.

Comment 5: *While Figures 2 and 3 illustrate the presence of β-catenin isoforms with distinct molecular weights across variants, it would be informative to also include quantitative data on expression levels for each variant.*

Our answer: **Following the reviewer suggestion, we have incorporated a new supplementary Fig. S1 showing quantitative data on the expression levels of the b-catenin variants.**

Comment 6: *The x-axis in the right panel of Figure 7A presents treatment groups, whereas other figures use gene variants. To facilitate comparison across figures, a uniform format for data presentation is recommended.*

Our answer: **Figure 7A has been re-arranged so all x-axis show gene variants.**

Comment 7: *The rationale for analyzing only N-terminal β-catenin in Figure 5 should be clarified. Was C-terminal detection not informative?*

Our answer: **Figure 5A shows analysis of variants using sequentially both C-terminal and N-terminal anti-β-catenin antibodies, which allows the detection of both C-terminal and N-terminal truncated variants. Figure 5B shows analysis using N-terminal antibody, since the analysed variants lack β-catenin C-terminal portion.**

Comment 8: *Is G418 expected to induce translational readthrough universally across all PTCs in the tested variants?*

Our answer: **As a general rule, the readthrough response to readthrough-inducing agents mostly depends on the identity of the stop codon (following this order of efficiency: TGA>TAG>TAA). In addition, the nucleotide context around the stop codon is also important for readthrough efficiency. This is well documented by us and by others, and it is now mentioned in the text in more detail, citing appropriate references. In addition, we have added to Table 1 the identity of the premature stop codons present in the nonsense variants.**

Comment 9: *The study utilizes CHX, MG-132, and Y-320 to modulate β-catenin expression. It should be clarified whether these reagents act specifically on β-catenin or exert broader effects on global protein synthesis and degradation. If they are nonspecific, a discussion of potential off-target or compensatory mechanisms is warranted.*

Our answer: **We agree with the reviewer that the effects of the agents used to modulate β-catenin expression affect globally protein synthesis and degradation. Accordingly, a statement has been added to the Discussion section at this regard.**

Comment 10: *The inhibitory effect of CHX on β-catenin expression is evident in Figure 5A but appears absent for the R661 variant in Figure 7A. This inconsistency should be addressed.*

Our answer: **We thank the reviewer for mentioning this inconsistency. We have repeated experiments and re-evaluated data, which are now more consistent. Note that following Comment 6, we have also re-organized this figure.**

Comment 11: *Although G418 promotes β-catenin upregulation, this effect is observed primarily in the R474X variant. The manuscript would benefit from a more detailed discussion on why G418's efficacy is limited to certain variants and what factors might underlie this selectivity.*

Our answer: **The R474X variant harbors a TGA stop codon, which favors the readthrough efficiency (see our answer to Comment 8). We have added information and discussion related to this issue in the manuscript.**

Comment 11: *While MG-132, Y-320, and G418 show potential for enhancing β-catenin levels in specific variants, the translational potential of these compounds requires further discussion, especially in terms of specificity, toxicity, and feasibility for clinical use.*

Our answer: **We agree with the reviewer on the potential and caveats of the agents used in the manuscript in terms of therapy, and we have discussed more in detail this topic in the revised manuscript as suggested.**

We thank again to all the reviewers and editors for their time and valuable input, and hope that the revised version of our manuscript is now suitable for publication at PLOS Genetics,

Sincerely,

Caroline E. Nunes-Xavier, PhD
carolinenunesxavier@gmail.com
carolineelisabeth.nunes-xavier@bio-bizkaia.eus

Rafael Pulido, PhD
rpulidomurillo@gmail.com
rafael.pulidomurillo@bio-bizkaia.eus
